# Balancing Nature and Nurture: The Role of Biocontrol Agents in Shaping Plant Microbiomes for Sustainable Agriculture

**DOI:** 10.3390/microorganisms13020323

**Published:** 2025-02-02

**Authors:** Suzana Moussa, Lilach Iasur Kruh

**Affiliations:** The Biotechnology Engineering Department, Braude College of Engineering, Karmiel 2161002, Israel

**Keywords:** bacterial biocontrol agents, microbiome, fungal biocontrol agents, *Trichoderma*, *Bacillus*

## Abstract

Microbial communities in the plant environment are highly dynamic, with bacterial populations rapidly responding to changes. Numerous studies have examined how both inherent plant characteristics and environmental factors shape plant-associated microbiota. These factors determine which bacterial communities thrive and how they interact with plants; certain conditions favor beneficial bacteria, and others support pathogens. In this mini-review, we focus on an additional factor influencing plant microbiomes and their surrounding environments: the use of biocontrol agents. The increasing application of microbial inoculants and their metabolites as biocontrol strategies in agriculture has created a critical knowledge gap about the effects of introducing non-native bacterial species into natural plant ecosystems. The inoculation of plants and their environments with exogenous biocontrol microorganisms has the potential to alter microbial community diversity and composition, presenting both opportunities and challenges for sustainable agricultural practices.

## 1. Mini-Review

Microbial changes occur rapidly because of the quick response of bacteria to their environment. Many studies have examined the effects of various factors on the microbiota of the plant environment. In such cases, “nurture vs. nature” defines two key aspects of changes in bacterial population composition. Nature, in this context, refers to the inherent genetic makeup of plants and how it influences their interaction with bacteria. These interactions can increase nutrient uptake, disease resistance, and overall plant health. Thus, plant genotypes can significantly vary in their ability to benefit from bacterial associations, and strain-specific interactions have evolved. Indeed, the genetics of the plant are crucial in determining the assembly of their community composition [1,2,3].

Nurture, in this context, refers to environmental characteristics that influence the presence and activity of bacteria in and around plants. This includes factors such as soil composition, agricultural practices, and the use of fertilizers and pesticides. The environment plays a crucial role in determining which bacteria are present and how they interact with plants. For example, certain soil conditions may favor the growth of beneficial nitrogen-fixing bacteria while others may promote the growth of harmful pathogens [2,4]. Table 1 shows examples of various parameters that influence changes in the microbiota composition of various plant species, focusing on both nurture and nature parameters.

**Figure 1:**
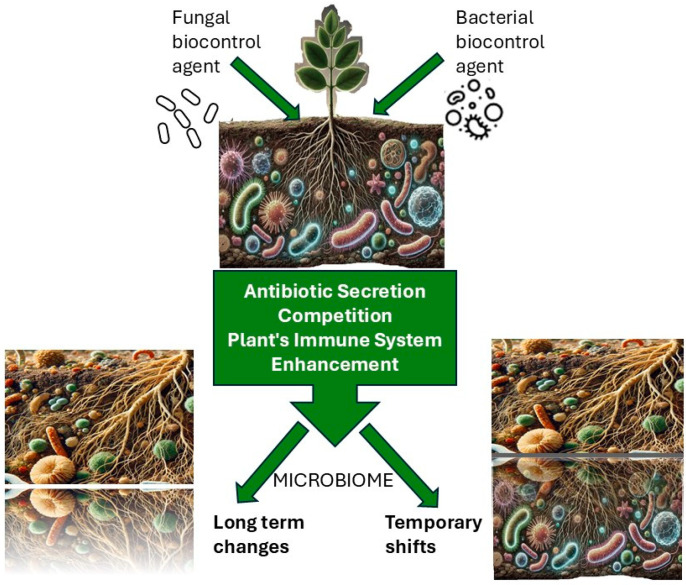
The mechanism by which the application of microbial biocontrol agents shapes the microbiome of a plant and its surrounding environment.

The present review focuses on an additional factor that may influence the plant microbiome and its surrounding environment: the use of biocontrol agents as part of agricultural practices (Figure 1). The growing use of microbial inoculants and their metabolites as a biological control strategy in modern agriculture [23,24] has resulted in a critical knowledge gap regarding the consequences of introducing non-native bacterial species into the natural ecosystems of plants. The inoculation of plants and their surrounding environments with beneficial exogenous biocontrol microorganisms can profoundly influence the diversity and composition of the microbial community [25,26,27], as was demonstrated by applying mycorrhizal fungi that changed the communities and activity of rhizosphere microorganisms [28,29,30].

Studies on various fungal biocontrol agents have shown that these organisms can significantly influence soil and plant microbial communities, ranging from minimal and transient to significant effects, often depending on factors such as soil depth, time since application, and the presence of organic amendments. For example, biomarker fatty acids representing the soil microbial community of tomato plants were affected by the fungal biocontrol *Clonostachys rosea* and *Glomus intraradices*. Ravnskov et al. [31] demonstrated that *G. intraradices* increased the prevalence of most groups of microorganisms, including gram-negative, gram-positive, Actinomycetes, and fungi. The effect of *C. rosea* on soil microorganisms varied depending on organic matter availability. Supplementing with wheat bran boosted *C. rosea*’s population density while suppressing most other soil microorganism groups. By contrast, *C. rosea* enhanced the biomass of multiple bacterial groups without wheat bran.

Other biocontrol fungi like *Beauveria bassiana* were shown to enhance both bacterial and fungal diversity, enriching the abundance of beneficial bacterial genera, like *Burkholderia* and *Pseudomonas*, and increasing the overall microbiome complexity [32]. The effect of the endophytic fungi Epichloë was more pronounced in the root endosphere than in the rhizosphere, as observed in the *Leymus chinensis* microbiota. This biocontrol agent affects more significantly fungal communities than bacterial ones, increasing the abundance of three fungal families—Thelebolaceae, Herpotrichiellaceae, and Trimorphomycetaceae [33].

*Trichoderma* species are widely used as biocontrol fungi [34], and their impact on microbial communities varies depending on plant genotype and environmental conditions (Table 2). Recent studies on *Trichoderma* species as biocontrol agents revealed that, although their application may have had minimal effect on overall microbial diversity, they significantly influenced microbial community composition, particularly affecting bacteria and fungi differently. For example, in peanut and bean crops, the strains *T. harzianum* ESALQ-1306 and *T. asperellum* BRM-29104 induced noticeable shifts in bacterial and fungal community structures. Despite limited changes in diversity metrics, these strains introduced unique genera in bacterial communities, with *T. harzianum* ESALQ-1306 yielding the highest number of unique bacterial taxa whereas fungal diversity was less affected [35]. By contrast, analysis in vineyards showed that *T. atroviride SC1* caused only short-term changes in microbial communities, with a greater effect on fungal than bacterial rhizosphere communities, as evidenced by lower alpha diversity owing to fungal dominance [36,37]. This pattern was confirmed in a strawberry phyllosphere and ginger roots augmented with *T. harzianum T22* and *T. Atroviride HB20111*, respectively, which altered fungal composition and diversity but left bacterial communities unchanged [38,39].

These findings suggest that fungal biocontrol agents varied in their capacity to reshape microbial community structure and that these alterations appear to be strain-specific to both the fungus used and the plant species involved.

The application of prokaryotes to agricultural crops has shown varying effects on the rhizosphere community composition. In some cases, the influence of an applied bacterium persists from seed to mature plant, as demonstrated by *Lysobacter antibioticus* 13-6, which was applied as a seed coating and significantly influenced the bacterial community composition of mature plant rhizosphere during a field trial [44] (Table 3). However, recent studies across various agricultural systems indicate that most bacterial biocontrol agents generally cause only temporary shifts in microbiota, without leading to long-term disruptions in the equilibrium of rhizosphere and soil community composition (Table 3).

In various soil types, ranging from acidic agricultural soil to clay loam, *Bacillus subtilis* PTA-271 has been shown to temporarily alter microbial community composition while preserving overall ecosystem stability [36]. In zucchini crops, *B. subtilis* effectively controlled *Phytophthora capsici* in both natural and artificially infested soils, achieving disease reduction rates of between 31.9% and 60.1% while maintaining rhizosphere microbial balance [49]. In addition to soil type, plant genotype plays a crucial role in the effect of exogenous bacteria on the plant system; this is exemplified by cultivar-dependent effects in sweet potatoes after applying a *Bacillus* sp. biofertilizer [45].

Another biocontrol-based *Bacillus* species, *B. velezensis* T-5, modifies tomato root exudates, leading to shifts in soil microbiota and changes in bacterial community diversity indices, including the Shannon evenness index, inverse Simpson diversity index, and Shannon diversity index. In addition, this bacterium caused a significant increase in the relative abundance of Bacteroidetes, Alphaproteobacteria, and Verrucomicrobia as well as a decrease in the relative abundance of Actinobacteria and Candidatus Saccharibacteria. At the family level, T-5-inoculated root exudates significantly increased the relative abundance of Geodermatophilaceae, Sphingomonadaceae, Mycobacteriaceae, Methylobacteriaceae, Chitinophagaceae, Bradyrhizobiaceae, Oxalobacteraceae, and Pseudonocardiaceae. Conversely, they decreased the relative abundance of families such as Acetobacteraceae, Dermabacteraceae, and Micrococcaceae [46]. Similarly, in tomato crops, *B. amyloliquefaciens* SN16-1 induced temporary increases in specific bacterial genera like *Pseudomonas* and *Massilia* while decreasing *Arenimonas*, *Brevundimonas*, and *Nocardioides*, with communities returning to baseline after 40 days [47]. Another strain of *B. amyloliquefaciens*, named WS-10, influences the diversity and composition of rhizosphere microbial communities of tobacco—elevating Simpson and Shannon diversity indices in both fungi and bacterial communities. In addition, this biocontrol strain enhanced the relative abundance of Gemmatimonadetes and Cyanobacteria phyla [48]. This pattern of transient impact was also observed in cucumber and barley systems treated with *Pseudomonas fluorescens* 2P24 strain [50] and DR54 strain [51], respectively, where initial changes in bacterial (mainly an increase in Gram-negative) or fungal populations reverted to their original states within a month. These findings suggest that bacterial biocontrol agents can effectively fulfill their protective role without causing a lasting disruption to soil microbiota, demonstrating their potential as environmentally sustainable crop protection tools. Furthermore, certain bacterial strains can demonstrate a strain-specific influence. The application of the bacterial biocontrol agent *Pseudomonas fluorescens* CHA0 to the rhizosphere of mungbean significantly increased populations of *Aspergillus niger* and *Trichoderma viride* while suppressing *Fusarium oxysporum*. Hence, although all major fungal species were commonly isolated from both treated and untreated rhizospheres, certain species were specifically promoted or suppressed in *Pseudomonas*-treated soils. Additionally, the effect of this biocontrol strain, as indicated by direct fungal counts, showed that the total number of species and genera was significantly lower (*p* < 0.01) in CHA0-treated soils than in the controls [52]. Another example is the application of three *Streptomyces* strains as bacterial biocontrol agents to wheat-modulated root microbiome, decreasing *Paenibacillus* abundance while increasing other bacterial and fungal OTUs such as *Exophiala*, *Phaeoacremonium*, and *Xylariaceae*. Yet, not all *Streptomyces* strains exhibited the same effect on the plant microbiome, suggesting strain-specific interactions. The data revealed that sampling time exercised a stronger influence (*p* < 0.001) on the richness and composition of microbial communities in roots and rhizosphere samples than other factors like biocontrol treatment and *Rhizoctonia* soil level [55].

Endophytic microorganisms form close interactions with their host by inhabiting the inner tissues of the plant. Therefore, it was expected that bacterial biocontrol agents based on endophytic bacteria would significantly alter the plant microbiome. Indeed, the effect of endophytic biocontrol agents *Serratia plymuthica* 3Re4-18 and *Pseudomonas trivialis* 3Re2-7 was found to be more pronounced in the root endosphere than in the rhizosphere, as observed in lettuce [54]. However, the patterns exhibited by various endophytic bacterial strains were inconsistent. Some studies reported increased microbial diversity following biocontrol agent application [45]. For example, when applied to potato plants, *Pseudomonas putida* P9, a biocontrol endophytic bacterium, demonstrated effective colonization of both the rhizoplane and endosphere, persisting through different growth stages and altering the abundance of certain bacterial groups like *P. azotoformans*, *P. veronica*, and *P. syringae* [53]. Other research, however, found only minor, short-term effects [54], attesting to the complexity of these interactions.

Only a handful of studies combined the application of bacterial and fungal biocontrol agents to examine whether they significantly affected microbial communities and diversity. In the rhizosphere, the combined application of *Trichoderma harzianum* and *Bacillus subtilis* demonstrated significant effects on bacterial communities at both phylum and genus levels, with notable increases in Acidobacteria, Planctomycetes, Chloroflexi, and Gemmatimonadetes, while decreasing Actinobacteria abundance. This treatment enhanced overall bacterial diversity, intensifying effects at higher BCA dosages [56]. Conversely, in the phyllosphere, the application of various BCAs (*B. amyloliquefaciens FZB42*, *T. harzianum T22*, and *Beauveria bassiana ATCC 74040*) showed differential impacts: fungal composition and diversity were significantly altered at the class level while bacterial communities remained largely unaffected by any of the applied BCAs [38]. A similar trend was observed where *Trichoderma* dominated the fungal community composition, and the diversity of treatments was reduced where it was applied, both alone and in combination with *Bacillus subtilis*. By contrast, treatments with *B. subtilis* alone did not affect bacterial or fungal diversity or community composition [36].

## 2. Summary

The growing demand for green technologies in agricultural practices demonstrates the importance of using beneficial microorganisms. Nevertheless, introducing these exogenous microorganisms can influence the microbiome of soil and plants, potentially harming soil fertility and plant health in a long-term ecological perspective. This manuscript reviewed findings that shed light on the complex interactions between introduced biocontrol agents and native microbial communities in plants. The effects of these agents were found to vary significantly by strain, with each biocontrol agent uniquely altering the diversity and composition of the plant-associated microbiome. These findings indicate the need for tailored biocontrol strategies that carefully evaluate and account for the ecological impact of biocontrol microorganisms on their surrounding environment.

## Figures and Tables

**Table 1:** Parameters influencing plant microbiota composition focusing on abiotic factors and plant genotypes.

Plant Type	Environmental Conditions	Differences in Bacterial Populations	Reference
Mulberry cultivars	Seasonal variation	Endophytic bacterial communities varied seasonally and between mulberry cultivars	[5]
Grapevine cultivars	Growing region, plant genotype, plant growth stage	Bacterial microbiomes differed based on region, cultivar, and growth stage	[6]
Cotton	Spatiotemporal variation	Endophytic communities showed spatial and temporal shifts in cotton roots	[7]
Mustard plant	Climate	Climate caused divergence in plant-microbiome interactions affecting phenology	[8]
Soybean	Soil, plant genotype	Soil microbiome and plant genotype shaped rhizosphere microbiome assembly	[9]
Sleepy grass	Plant genotype	Plant population and genotype overrode effects of endophyte on growth/drought	[10]
Barley and grass plants	Soil pH	Alkaline soil pH affected microbiomes of bulk soil, rhizosphere, and endosphere	[11]
Grapefruit	Substrate pH	Substrate pH influenced nutrient uptake and rhizosphere microbiome	[12]
Crofton weed	Soil pH	Soil pH affected growth, soil nutrients, and rhizosphere microbiome	[13]
Lettuce, wheat, oat	Soil composition	Rhizosphere microbiome stability depended on plant type and soil	[14]
Cotton	Soil type, plant genotype, development	Rhizosphere microbiome varied with soil, genotype, and development stage	[15]
Lettuce	Soil type	Soil type affected the rhizosphere microbiome of field-grown lettuce	[16]
Arabidopsis	Plant development stage	Rhizosphere microbiome assembly was affected by plant development stage	[17]
Arabidopsis	Climate, geographic distance	Climate caused rhizosphere microbiome variation in distant populations	[18]
Drummond’s rockcress	Host genotype, plant age	Host genotype and age shaped leaf and root microbiomes	[19]
Olive	Plant genotype	Plant genotype shaped microbiomes of fruits, leaves, and soil	[20]
Wheat	Plant genotype, plant development stage	Wheat genotype effect was more evident in roots and it varied over time	[21]
Oak, olive, grapevine	Temperature	Temperature caused seasonal succession of endophytic communities	[22]

**Table 2:** The effect of *Trichoderma* strains on microbial community dynamics and diversity in different crop rhizospheres.

Trichoderma Strains	Crop/System	Analytical Methods	Microbial Community Impact and Key Findings	Reference
*T. atroviride SC1*	Grapevines	qPCR, BIOLOG Microtiter™ GN2 plates, NGS	Short-term shifts in microbial communities; greater impact on fungal than bacterial communities, with significantly lower alpha diversity due to fungal dominance.	[36,37]
*T. atroviride HB20111*	American ginseng	NGS	Alterations in bacterial communities of the cortex; fungi more affected in plant tissues, with enhanced abundance of Novosphingobium and Pseudogymnoascus; stronger impact on plant-associated microbes.	[39]
*T. harzianum ITEM 3636*	Peanut	DGGE, NGS	Changes in microbial community composition; minimal impact on diversity, but shifts in community composition and functionality.	[40]
*T. harzianum ESALQ-1306*	Beans	NGS	Significant changes in bacterial and fungal community composition in the rhizosphere; control treatment maintained highest fungal diversity; *T. harzianum* ESALQ-1306 produced the most unique taxa.	[41]
	Common Beans	NGS	Significant changes in endophytic fungal diversity and dominance; fungal diversity and dominance varied at the phylum and family level.	[35]
*T. harzianum R3P2*	Tobacco	BIOLOG Microtiter™ GN2 plates	Changes in soil microbial community and metabolic diversity; short-term shifts in microbial community.	[42]
*T. harzianum T22*	Strawberries	Pyrosequencing of ITS ribosomal RNA and 16S RNA	Altered fungal composition and diversity in phyllosphere; increased abundance of Sordariomycetes and decreased Dothideomycetes; no effect on bacterial diversity.	[38]
*Trichoderma asperellum MSCL 309*	Soil sample	Biolog EcoPlate, direct count	Did not significantly affect the metabolic diversity of the community but changed the utilization of carbohydrates, complex carbon compounds, and organic phosphorus compounds.	[43]

**Table 3:** Effect of bacterial biocontrol strains on microbial community dynamics and diversity across different crop rhizospheres.

Biocontrol Genus	Biocontrol Strain	Crop	Effect on Microbial Community	Duration of Effect	Reference
*Lysobacter*	antibioticus 13-6	Maize	Significant increase at the rhizosphere relative abundance of Gammaproteobacteria, Gemmatimonadetes, and Bacteroidetes at the phylum level, as well as Streptomyces, Lysobacter, and Nitrospira at the genus level.	From seed coating until mature plant	[44]
*Bacillus*	*subtilis*	Grapevine	Successful establishment in clay loam but minimal alteration of existing bacterial microbiome.	Temporary, no significant long-term disruption.	[36]
	sp. biofertilizer (OYK)	Sweet Potato	Changed endophytic bacterial composition in a cultivar-dependent manner, with increased Shannon diversity index.	Transient impact on microbial diversity	[45]
	*velezensis T-5*	Tomato	Altered root exudates, increased diversity indices (Shannon evenness, inverse Simpson, Shannon diversity), increased Bacteroidetes, Alphaproteobacteria, and Verrucomicrobia, while decreasing Actinobacteria.	Significant short-term effects.	[46]
	*amyloliquefaciens SN16-1*	Tomato	Increased *Pseudomonas* and *Massilia* while decreasing *Arenimonas*, *Brevundimonas*, and *Nocardioides*.	Transient (short-term, community reversion within 40 days).	[47]
	*amyloliquefaciens*, WS-10	Tabacco	Changed both diversity indices and bacterial and fungal community composition	Examined one time at the end of a pot experiment	[48]
	*subtilis*	Zucchini	Controlled *Phytophthora capsici*, disease reduction rates of 31.9% to 60.1% while maintaining microbial balance in the rhizosphere.	Temporary, maintained microbial balance	[49]
*Pseudomonas*	*fluorescens 2P24*	Cucumber	Temporary changes in bacterial populations mainly increased Gram-negative bacteria.	Transient, returned to baseline in about one month.	[50]
	*fluorescens DR54*	Barley	Temporary shifts in microbial populations.	Transient, reversion within a month.	[51]
	*fluorescens CHA0*	Mungbean	Reduced fungal diversity, increased *Aspergillus niger* and *Trichoderma viride* while suppressing *Fusarium oxysporum*.	Long-term, strain-specific changes observed.	[52]
	*putida P9*	Potato	Altered abundance of *Pseudomonas azotoformans, Pseudomonas veronii*, and *Pseudomonas syringae* in rhizoplane and endosphere.	Persisted through different growth stages.	[53]
	*trivialis 3Re2-7*	Lettuce	A pronounced effect was found in root endosphere.	Minor short-term effects observed.	[54]
*Streptomyces*	*Different strains*	Wheat	Modulated root microbiome, decreased *Paenibacillus*, increased *Exophiala, Phaeoacremonium* and *Xylariaceae*, with time-dependent microbial changes.	Temporal dynamics played a crucial role.	[55]
*Serratia*	*plymuthica 3Re4-18*	Lettuce	More pronounced effects in root endosphere compared to rhizosphere, altering microbial composition.	Minor short-term effects observed.	[54]
